# Maternal Factors and Adverse Perinatal Outcomes in Women with Preeclampsia in Maceió, Alagoas

**DOI:** 10.5935/abc.20150150

**Published:** 2016-02

**Authors:** Alane Cabral Menezes de Oliveira, Arianne Albuquerque Santos, Alexandra Rodrigues Bezerra, Amanda Maria Rocha de Barros, Myrian Cicyanne Machado Tavares

**Affiliations:** 1 Universidade Federal de Alagoas, Maceió, AL - Brazil; 2 Hospital Universitário Professor Alberto Antunes, Maceió, AL - Brazil

**Keywords:** Risk Factors, Hypertension, Pre-Eclampsia, Pregnant Women, Perinatal Care

## Abstract

**Background:**

Preeclampsia has been associated with several risk factors and events. However, it still deserves further investigation, considering the multitude of related factors that affect different populations.

**Objective:**

To evaluate the maternal factors and adverse perinatal outcomes in a cohort of pregnant women with preeclampsia receiving care in the public health network of the city of Maceió.

**Methods:**

Prospective cohort study carried out in 2014 in the public health network of the city with a sample of pregnant women calculated based on a prevalence of preeclampsia of 17%, confidence level of 90%, power of 80%, and ratio of 1:1. We applied a questionnaire to collect socioeconomic, personal, and anthropometric data, and retrieved perinatal variables from medical records and certificates of live birth. The analysis was performed with Poisson regression and chi-square test considering p values < 0.05 as significant.

**Results:**

We evaluated 90 pregnant women with preeclampsia (PWP) and 90 pregnant women without preeclampsia (PWoP). A previous history of preeclampsia (prevalence ratio [PR] = 1.57, 95% confidence interval [95% CI] 1.47 - 1.67, p = 0.000) and black skin color (PR = 1.15, 95% CI 1.00 - 1.33, p = 0.040) were associated with the occurrence of preeclampsia. Among the newborns of PWP and PWoP, respectively, 12.5% and 13.1% (p = 0.907) were small for gestational age and 25.0% and 23.2% (p = 0.994) were large for gestational age. There was a predominance of cesarean delivery.

**Conclusion:**

Personal history of preeclampsia and black skin color were associated with the occurrence of preeclampsia. There was a high frequency of birth weight deviations and cesarean deliveries.

## Introduction

The hypertensive syndromes of pregnancy deserve special attention in the scenario of global and national public health. These syndromes are currently the first cause of maternal mortality in Brazil, affecting approximately 5 to 17% of all pregnant women. Due to their severity, they are among the most important causes of hospitalization in intensive care units (ICU).^1-7^

Preeclampsia (PE) is a disorder that results from poor placental perfusion and endothelial dysfunction with an increase in blood pressure levels and proteinuria after the 20^th^ week of gestation.^1,8^ The occurrence of PE is associated with an increased risk of adverse events (placental abruption, acute renal failure, and cerebral hemorrhage, among others) and unfavorable perinatal outcomes (low birth weight [LBW], fetal macrosomia [FM], low Apgar score at 1 and 5 minutes, neonatal infection, meconium aspiration syndrome, and prematurity, among others).^9,10^

Several risk factors associated with PE have been described in the literature, including first delivery, extremes of reproductive age, inadequate pregestational or gestational nutrition, inappropriate weight gain, unfavorable socioeconomic conditions, presence of chronic diseases, and personal and / or family history of PE, among others. According to some authors, the incidence of the disease deserves more investigation, considering the multitude of factors modifying its risk in different geographic areas, since some factors are similar between populations, whereas others are related to the geographic area and ethnicity of the studied cohort.^11-19^

Decrease in maternal and infant mortality is one of the targets for worldwide poverty reduction until 2015 (Millennium Development Goals [MDGS-2009/2012]).^20^ Despite the importance of PE and the potential to prevent most deaths and associated complications, there are no studies about this subject in Maceió. Considering that, the present study aimed at comparing the maternal factors and adverse perinatal outcomes in pregnant women with PE and normotensive pregnant women receiving care in the public health network of Maceió. This analysis intends to direct the strategies to reduce PE and its complications.

## Methods

This was a prospective cohort study carried out in 2014 with pregnant women with PE seen at *Hospital Universitário Professor Alberto Antunes* (HUPAA, a reference center for high-risk pregnancy in the state) and normotensive pregnant women undergoing prenatal care in Basic Health Units (*Unidades Básicas de Saúde*, UBS) in the city of Maceió, state of Alagoas.

The sample size was calculated with the software Epi Info, version 7.0, based on a PE prevalence of 17%,^7^ considering a confidence level of 90%, power of 80%, and ratio of 1:1 (exposed and not exposed). The estimated sample size was 178, comprising 89 pregnant women with preeclampsia (PWP) and 89 pregnant women without preeclampsia (PWoP).

The inclusion criteria were residence in Maceió and prenatal care received at HUPAA or in a UBS in the city. Pregnant women not residing in the city, with restricted mobility, not receiving care at HUPAA, or who were not undergoing prenatal care in a UBS in the city were not included in the study.

After selecting the participants, we applied a standardized questionnaire which had been previously tested by the research group, and collected data on socioeconomic (income, education level, and reported skin color), personal (personal and family history of PE, civil status, and parity), anthropometric (pregestational weight, current weight, and height), and perinatal variables (gestational age [GA] at birth, weight and length of the newborn [NB] at birth, gender of the NB, type of delivery, and Apgar score at 1 and 5 minutes). The latter data were collected after birth from medical records and / or certificates of live birth.

The diagnosis of PE was confirmed by data retrieved from medical records (medical opinion) in the presence of hypertension (systolic blood pressure ≥ 140 mmHg or diastolic arterial pressure ≥ 90 mmHg) and proteinuria (urinary protein > 300 mg/24h) after the 20th week of pregnancy.^8^

To assess the maternal nutritional status, we measured the weight and height of the women with a digital scale and a stadiometer, and used cutoff values established by Atalah et al.^21^ and recommended by the Brazilian Ministry of Health.^22^ We also recorded the weight gain during pregnancy adjusted for the GA at the moment of the interview, considering the weight goal recommended by the Institute of Medicine (IOM).^23^

The GA of the newborn at birth was classified according to the criteria proposed by the World Health Organization (WHO):^24^ preterm (GA < 37 weeks), term (GA between 37 and 42 weeks), and post-term (GA > 42 weeks). Weight and length at birth were interpreted according to the new WHO charts,^25^ or Fenton charts for NBs with GA < 33 weeks.^26^ The cutoff values for both charts were considered in percentiles according to international standards. The NBs with weight below the 3rd percentile were classified as small for gestational age (SGA), those between the 3rd and 97th percentiles as appropriate for gestational age (AGA), and those with weight above the 97th percentile as large for gestational age (LGA). The same cutoff values were considered to categorize the length at birth. The status of the NB after birth was evaluated with the Apgar score at 1 and 5 minutes, in which values ≤ 6 for both minutes characterized risk for the NB.^27^

The data were processed using the software Stata, version 13.0, adopting a confidence level of 95% (α = 0.05). We used Poisson regression with robust variance estimate to identify maternal factors associated with PE, and tested in the adjusted model the independent variables that in the crude regression analysis showed significance below 20% (p < 0.20). The magnitude of the associations between the outcome variable and the independent variables were expressed as prevalence ratio (PR) and 95% confidence interval (95%CI). The outcome variable of the analyses was the presence or absence of PE. The independent variables were maternal age (≤ 19 years, 20 to 34 years, or ≥ 35 years), family income (< 1 minimum wage or ≥ 1 minimum wage), maternal years of education (< 4 years or ≥ 4 years), reported color of the skin (white or brown / black), presence of a stable relationship (yes or no), first pregnancy (yes or no), personal history of PE (yes or no), family history of PE (yes or no), gestational nutritional status according to the body mass index (BMI; low weight, eutrophic, overweight, or obese), and weight gain during pregnancy (insufficient, adequate, or excessive).

We used the chi-square test to characterize the perinatal variables, aiming to compare the frequencies between the two groups (PWP and PWoP), with results expressed as odds ratio (OR) and 95% CI.

The study was approved by the Ethics and Research Committee of *Universidade Federal de Alagoas* (UFAL), with the process number 341.953.

## Results

We evaluated 90 PWP and 90 PWoP, with mean ages of 25.8 ± 6.7 years and 24.1 ± 6.2 years, respectively. In all, 17.8% of the PWP and 27.8% of the PWoP were adolescents (p = 0.096), 43.3% and 45.5%, respectively, had low education level (p = 0.433), and 30.0% and 24.4%, respectively, had low income (p = 0.407). Black skin color was reported by 16.7% of the PWP and 10.0% of the PWoP (p = 0.194), whereas 28.9% and 8.9%, respectively, had a family history of PE (p = 0.000), and 38.9% and 1.11%, respectively, had a personal history of PE (p = 0.000). Obesity rates were 40.1% and 13.0%, respectively (p = 0.000), and rates of excessive gestational weight gain were 34.5% and 16.7%, respectively (p = 0.013) (Table 1).

**Table 1:** Distribution of PE, and crude (95% CI) PRs according to socioeconomic and personal variables, and anthropometric measurements of pregnant women attending the public health network of the city of Maceió, Alagoas, 2014

**Variable**	**PWP (n = 90)**	**PWoP (n = 90)**	**Crude PR (95% CI)**	p*
**Age group (years)**				
≤ 19	16 (17.8)	25 (27.8)	0.91 (0.82-1.02)	0.096
20-34	66 (73.3)	56 (62.2)	1.00	
≥ 35	8(8.9)	9 (10.0)	0.95 (0.81-1.13)	0.592
**Years of education**				0.433
<4	39 (43.3)	41 (45.5)	0.98 (0.95-1.02)	
≥ 4	51 (56.7)	49 (54.5)	1.00	
**Income (Brazilian Real)**				0.407
< 1 minimum wage	27 (30.0)	22 (24.4)	1.04 (0.93-1.17)	
≥ 1 minimum wage	63 (70.0)	68 (75.6)	1.00	
**Skin color (reported)**				0.194
Black	15 (16.7)	9 (10.0)	1.11 (0.95-1.28)	
White / dark	75 (83.3)	81 (90.0)	1.00	
**Stable relationship**				0.880
No	38 (42.2)	37 (41.1)	1.01 (0.91-1.11)	
Yes	42 (57.8)	53 (58.9)	1.00	
**Family history of PE**				0.000
Yes	26 (28.9)	8 (8.9)	1.26 (1.11-1.43)	
No	64 (71.1)	82 (91.1)	1.00	
**Personal history of PE**				0.000
Yes	35 (38.9)	1 (1.11)	1.62 (1.54-1.70)	
No	55 (61.1)	89 (98.9)	1.00	
**First pregnancy**				0.762
Yes	36 (40.0)	38 (42.2)	0.98 (0.89-1.09)	
No	54 (60.0)	52 (57.8)	1.00	
**Gestational nutritional status**				
Low weight	13 (14.4)	15 (16.7)	0.97 (0.85-1.10)	0.678
Eutrophy	22 (24.4)	39 (43.3)	1.00	
Overweight	19 (21.1)	24 (26.7)	0.95 (0.85-1.06)	0.375
Obesity	36 (40.1)	12 (13.3)	1.27 (1.14-1.42)	0.000
**Gestational weight gain**				
Insufficient	41 (45.5)	45 (50.0)	0.98 (0.89-1.08)	0.705
Adequate	18 (20.0)	30 (33.3)	1.00	
Excessive	29 (34.5)	15 (16.7)	1.16 (1.03-1.30)	0.013
No information	2	---	---	---

PWP: pregnant women with preeclampsia; PWoP: pregnant women without preeclampsia; PE: preeclampsia; PR: prevalence ratio; 95% CI: 95% confidence interval.

* Crude logistic regression, with p < 0.05 considered significant.

Table 2 shows the PE-associated factors that were included in the adjusted Poisson regression model. There was an association between PE and previous history of PE (PR = 1.57, 95% CI 1.47 - 1.67, p = 0.000) and black skin color (PR = 1.15, 95%CI 1.00 - 1.33, p = 0.040). The variables age ≤ 19 years, family history of PE, obesity according to BMI, and excessive weight gain were included in the adjusted model after reaching a p value within the limits established to maintain a variable in the model (p < 0.2).

**Table 2:** Factors associated with PE included in the multivariate model, Maceió, Alagoas, 2014

**Variable**	**Adjusted PR (95%CI)**	p*
Personal history of PE	1.57 (1.47-1.67)	0.000
Obesity by current BMI	1.10 (0.97-1.24)	0.115
Family history of PE	1.10 (0.98-1.24)	0.078
Excessive weight gain	1.08 (0.94-1.18)	0.324
Age ≤ 19 years	0.93(0.85-1.01)	0.090
Black skin color	1.15 (1.00-1.33)	0.040

PE: preeclampsia; BMI: body mass index; PR: prevalence ratio; 95% CI: 95% confidence interval.

* Poisson regression, with p < 0.05 considered significant.

Table 3 shows the perinatal outcomes of the studied cohort. These results excluded two PWP due to neonatal mortality, and five PWoP due to one case of spontaneous abortion, two cases of neonatal mortality, and two cases that were lost to follow up. The PWP and PWoP groups presented, respectively, 6.8% and 4.7% of preterm labors (OR = 1.46, 95% CI 0.39 - 5.38, p = 0.565), 12.5% and 13.1% of SGA NBs (OR = 0.95, 95% CI 0.39 - 2.32, p = 0.907), 25.0% and 26.2% of LGA NBs (OR = 0.99, 95% CI 0.50 - 1.97, p = 0.994), and 56.0% and 30.8% of NBs with increased birth length (OR = 2.96, 95% CI 1.56 - 5.61, p = 0.001). Cesarean delivery was the most frequent delivery route in both groups (58.0% and 53.9%, respectively). An Apgar score ≤ 6 at 1 minute was observed in 11.1% and 3.4% of the NBs in each group, respectively, and at 5 minutes in 6.7% and 3.4%, respectively.

**Table 3:** Perinatal results of pregnant women with PE attending the public health network in the city of Maceió, Alagoas, 2014

**Variable**	**PWP (n = 88)**	**PWoP (n = 85)**	**Crude OR (95%CI)**	p*
**Gestational age at delivery**				
Preterm	6 (6.8)	4 (4.7)	1.46 (0.39-5.38)	0.565
Term	75 (85.2)	73 (85.9)	0.87 (0.37-2.06)	0.751
Post-term	7 (8.0)	7 (9.4)	0.80 (0.26-2.50)	0.707
**Delivery route**				0.611
Cesarean	51 (58.0)	46 (53.9)	0.85 (0.47-1.57)	
Vaginal	37 (42.0)	38 (46.1)		
**NB gender**				1.000
Female	44 (50.0)	42 (50.0)	1.00 (0.55-1.82)	
Male	44 (50.0)	42 (50.0)		
**NB birth weight**				
SGA	11 (12.5)	11 (13.1)	0.95 (0.39-2.32)	0.907
AGA	55 (62.5)	51 (60.7)	1.07 (0.58-1.99)	0.810
LGA	22 (25.0)	22 (26.2)	0.99 (0.50-1.97)	0.994
**NB length at birth**				
Low	1 (1.1)	5(6.4)	0.17 (0.02-1.47)	0.069
Adequate	37 (42.9)	49 (62.8)	0.43 (0.23-0.80)	0.008
Increased	50 (56.0)	24 (30.8)	2.96 (1.56-5.61)	0.001
No information	---	7		
**NB Apgar score at 1 minute**				0.119
≤ 6	5 (11.1)	2 (3.4)	3.56 (0.66-19.29)	
≥ 7	40 (88.9)	57 (96.6)		
No information	43	26		
**NB Apgar score at 5 minutes**				0.198
≤ 6	3 (6.7)	2 (3.4)	4.07 (0.41-40.53)	
≥ 7	42 (93.3)	57 (96.6)		
No information	43	26		

PE: preeclampsia; PWP: pregnant women with preeclampsia (two cases of neonatal mortality); PWoP: pregnant women without preeclampsia (one case of spontaneous abortion, two of neonatal mortality, and two lost to follow up); NB: newborn; AGA: appropriate for gestational age; SGA: small for gestational age; LGA: large for gestational age; OR: odds ratio; 95%CI: 95% confidence interval.

* Chi-square test, with p < 0.05 considered significant.

## Discussion

The results of this study show that a personal history of PE is associated with a new occurrence of PE in a later pregnancy. Similarly, a study with a cohort of pregnant women in Sweden has shown that a previous history of PE also conferred greater risk for the disease, with an incidence of PE of 14.7% in women who had presented PE in the first pregnancy and 31.9% in those who had presented the disease in two previous pregnancies.^11^ Additionally, a survey conducted in the south of Brazil by Dalmaz et al.^14^ found a higher risk of PE in pregnant women with family history of the disease.

Women who develop PE have a higher risk of recurrence of the disease in future pregnancies, and often have a family history of the disease, which suggests the involvement of genetic factors. Studies have shown the importance of maternal genes in the development of PE, including genetic mutations (i) in the glu298Asp of the nitric oxide synthase, increasing the peripheral vascular resistance and (ii) in the factor V Leiden related to the blood coagulation system. However, the results regarding the genetic etiology of pre-eclampsia are not conclusive.^28^

Individuals with black skin color seem to have a hereditary defect in cellular uptake and renal transport of sodium and calcium, which can be attributed to a sodium-sparing gene that favors cellular sodium influx and calcium efflux, thus favoring the onset of hypertension.^29^ In a case-control study carried out with pregnant women in the state of Goiás, a skin color different than white was an independent risk factor for PE,^18^ corroborating the findings of the present study. This can be explained by the fact that black women present a higher incidence of chronic hypertension, which increases the incidence of PE superimposed to hypertension.

Studies have demonstrated a relationship between worse socioeconomic conditions and a higher incidence of PE since these conditions may be associated with situations of stress and worse nutritional status.^30^ In the present study, there was no association between unfavorable socioeconomic conditions, such as low income and low education level, and occurrence of PE. This can be explained by the homogeneity of the cohort, which included only pregnant women receiving care in the public health network of Maceió. This city, the largest in the state of Alagoas, has a Human Development Index (HDI) of 0.721, considered the worst in the country when taking into account income, longevity, and education criteria.^31^

This research also found no association between maternal nutritional status and occurrence of PE, despite higher frequencies of obesity and excess weight gain in PWP when compared with PWoP. In contrast, the international, multicenter, and epidemiological study HAPO^19^, which included 15 centers in nine countries, concluded that a high maternal BMI is associated with a higher frequency of the disease. Additionally, Seabra et al.,^32^ studying pregnant women attending the PE clinic in a public maternity in Rio de Janeiro, also found an increased risk of PE in women with overweight and obesity. The mechanisms for the predisposition of women with excess weight to PE are not yet entirely clear, but the hypotheses include an increased inflammatory response (as a consequence of an increased synthesis of proinflammatory substances by the adipose tissue, as well as some cytokines and C-reactive protein) inhibiting, for example, the nitric oxide synthase, reducing the availability of nitric oxide and causing vasoconstriction.^33^

Cesarean delivery (the predominating delivery route in this study) increases the risk of maternal complications, especially in pregnant women with severe PE. This increases the chances of hemorrhagic manifestations, and infections, hypertensive peaks, and also prolongs the length of hospital stay.^1-4^ The Technical Manual for High-Risk Pregnancies adopted by the Brazilian Health Ministry emphasizes that "high-risk pregnancy is not synonymous with cesarean delivery," since in many situations it is possible to induce labor aiming a vaginal delivery route, or even wait for its spontaneous onset.^4^ Still, the rate of cesarean deliveries in our study was well above that recommended by the WHO (< 15%).^34^

An observational and retrospective study with pregnant women with PE who underwent labor at *Maternidade Escola do Rio de Janeiro* observed a higher risk of NBs SGA, prematurity, neonatal infection, and meconium aspiration syndrome.^10^ In the present study, the occurrence of PE did not increase the chance of weight deviations (SGA and LGA) in the NBs, nor the frequency of preterm delivery compared with pregnancies without PE. It is important to highlight the high frequencies of these adverse perinatal outcomes in both groups (PWP and PWoP), particularly regarding deviations of birth weight, and births of NBs SGA (12.5% and 13.1%) and LGA (25% and 26.2%), since these outcomes contrast with national data (8.46% of the cases of LBW and 5.05% of FM) and data from the state of Alagoas (7.68% of the cases of LBW and 5.44% of FM).^35^

The high frequency of NBs LGA is noteworthy in this study. This fact may be due to a historical trend of nutritional transition, reflected by a higher incidence of increased birth weight (identified as a new and relevant advanced manifestation of this transition).^36^ Reinforcing these findings, a survey conducted in the northeast area of Brazil has identified an association between FM and excessive gestational weight gain.^37^

Studies have suggested a positive correlation between leptin concentration in the umbilical cord and GA, weight, length, and neonatal ponderal index, with pregnant women showing higher levels of this hormone when compared with non-pregnant ones, mainly those with excess body weight during pregnancy.^38^ This hormone also has an important role in regulating the sympathetic nervous system and, consequently, controlling blood pressure. In a case-control study, pregnant women with PE presented levels of leptin three times higher than normotensive ones.^39^ In the present study, pregnant women with PE had an almost three times greater chance of having a NB with increased length at birth when compared with normotensive ones, which could be explained by the presence of PE and excess weight in pregnant women with PE when compared with those with normal blood pressure, increasing the levels of leptin and leading to increased fetal growth.

In this study, most pregnant women with PE had NBs with Apgar scores at 1 and 5 minutes above the cutoff value, but the presence of the disease did not increase the frequency of these indexes. In contrast, Oliveira et al.,^10^ studying the perinatal repercussions of women who had undergone labor at *Maternidade Escola da Universidade do Rio de Janeiro*, found a higher risk of low Apgar scores at 1 and 5 minutes in women with a diagnosis of PE. However, a large part of the cohort in our study had no register of the Apgar score in the medical records or certificate of live birth, which limits the generalization of the results. According to Costa e Frias,^40^ several causes may be associated with poor filing of certificates of live birth, such as lack of clarity in the manual of instructions for completion of the form, and heterogeneity of the professionals responsible for this task. According to the results found in the present study, there was no association between PE and worse perinatal outcomes when compared with the absence of PE (pregnant women with normal blood pressure), with the exception of a greater frequency of birth of NBs with increased length. This result differs from most of those found in the literature. A likely cause for this finding was the lack of differentiation between mild and severe cases of PE, since the severe cases are those associated with the worst obstetric outcomes.^4^ Although the sample size was adequate to estimate the prevalence of the outcomes investigated in this study, it may not have had statistical power to identify associations between some variables, particularly those with lower prevalence in the studied population.

Even so, some of the adverse obstetric outcomes in this study, such as a predominance of cesarean delivery and NB weight deviations, have higher frequencies than those of recommended standards. This shows the great importance of prenatal and multiprofessional care during medical appointments in identifying risks, guaranteeing maternal nutritional support, and treating diseases to reduce obstetric and neonatal afflictions.

Limitations of this study include the large loss of information related to the Apgar score due to incomplete filing of medical records and / or certificates of live birth, emphasizing the importance of correct filing by the professionals.

## Conclusions

The occurrence of PE was associated with a maternal history of PE and black skin, and with a high frequency of birth weight deviations and cesarean delivery.
